# Sympathetic stimulation increases serum lactate concentrations in patients admitted with sepsis: implications for resuscitation strategies

**DOI:** 10.1186/s13613-021-00805-9

**Published:** 2021-02-05

**Authors:** Nikhil Jagan, Lee E. Morrow, Ryan W. Walters, Robert W. Plambeck, Tej M. Patel, Douglas R. Moore, Mark A. Malesker

**Affiliations:** 1 Division of Pulmonary & Critical Care, Creighton University School of Medicine, 7710 Mercy Road, Suite 410, Omaha, NE 68124 USA; 2 VA Nebraska-Western Iowa, Section of Pulmonary and Critical Care, Omaha, USA; 3 Division of Clinical Research & Evaluative Sciences, Creighton University School of Medicine, Omaha, USA; 4 Department of Internal Medicine, Washington University School of Medicine, St. Louis, USA; 5 Creighton University School of Pharmacy and Health Professions, Omaha, USA

**Keywords:** Sepsis, Lactic acid, Shock, Critical care outcomes, Resuscitation, Prognosis

## Abstract

**Background:**

Diametrically opposed positions exist regarding the deleterious effects of elevated lactate. There are data suggesting that it is a detrimental proxy for tissue hypoperfusion and anaerobic metabolism in sepsis and an alternative viewpoint is that *some* of the hyperlactatemia produced maybe adaptive. This study was conducted to explore the relationship between serum lactate levels, mean arterial blood pressure (MAP), and sympathetic stimulation in patients with sepsis.

**Methods:**

Retrospective analysis of prospectively collected clinical data from four community-based hospitals and one academic medical center. 8173 adults were included. Heart rate (HR) was used as a surrogate marker of sympathetic stimulation. HR, MAP, and lactate levels were measured upon presentation.

**Results:**

MAP and HR interacted to affect lactate levels with the highest levels observed in patients with low MAP and high HR (3.6 mmol/L) and the lowest in patients with high MAP and low HR (2.2 mmol/L). The overall mortality rate was 12.4%. Each 10 beats/min increase in HR increased the odds of death 6.0% (95% CI 2.6% to 9.4%), each 1 mmol/L increase in lactate increased the odds of death 20.8% (95% CI 17.4% to 24.2%), whereas each 10 mmHg increase in MAP reduced the odds of death 12.3% (95% CI 9.2% to 15.4%). However, HR did not moderate or mediate the association between lactate and death.

**Conclusions:**

In septic patients, lactate production was associated with increased sympathetic activity (HR ≥ 90) and hypotension (MAP < 65 mmHg) and was a significant predictor of mortality. Because HR, lactate, and MAP were associated with mortality, our data support the present strategy of using these measurements to gauge severity of illness upon presentation. Since HR did not moderate or mediate the association between lactate and death, criticisms alleging that lactate caused by sympathetic stimulation is adaptive (i.e., less harmful) do not appear substantiated.

## Introduction

Although the incidence, associated morbidity, attributable mortality, and overall costs related to sepsis vary widely, it is generally agreed that sepsis is ubiquitous and adversely affects patients [1–4]. Early identification of sepsis and aggressive restoration of peripheral perfusion are the cornerstones of management. To improve outcomes, sepsis bundles focusing on early resuscitation and early antibiotics have been implemented. Serial measures of lactate—a byproduct of anaerobic metabolism used as a surrogate marker for suboptimal perfusion—are increasingly advocated as a target of resuscitation in sepsis bundles. The 2016 Surviving Sepsis Campaign Guidelines endorse measuring lactate levels within the first hour of resuscitation and every two to four hours thereafter if the initial level is > 2.0 mmol/L. [5]

Increased reliance on lactate levels to guide clinical decision-making in sepsis is driven by studies associating elevated lactate with increased mortality in sepsis. One retrospective study found that lactate levels greater than 2.5 mmol/L correlated with a 28-day mortality of 16.9% [6]. The prognostic value of lactate was further emphasized when lactate-directed resuscitation led to lower Sequential Organ Failure Assessment (SOFA) scores, earlier cessation of inotropes, earlier weaning from mechanical ventilation, earlier intensive care unit (ICU) discharge, and a 9.6% absolute reduction in hospital mortality when compared to usual care. [7] Rising levels of lactate are also associated with increasing mortality, regardless of the presence/absence of shock. [8] Taken collectively, these data suggest that elevated lactate is a deleterious proxy for tissue hypoperfusion and anaerobic metabolism in sepsis. However, there are other etiologies for hyperlactatemia including reduced clearance (i.e., liver dysfunction in the setting of hypoperfusion) and medication administration (i.e., large volumes of lactated Ringer’s solution, excessive albuterol) [9–12].

An alternative viewpoint is that some of the hyperlactatemia produced during sepsis may be an adaptive response. Sepsis-related increases in metabolic rate and catecholamine levels increase glycolysis, glycogenolysis, gluconeogenesis, and reduce insulin release. This cascade ultimately results in increased pyruvate production with shunting to lactate production which can be used as an alternate energy source. The net effect is increased lactate production in the absence of tissue hypoxia—a form of Type B2 lactic acidosis [13, 14]. This line of thinking, espoused in several popular online blogs and podcasts, suggests that because not all of the etiologies for increased lactate seen are harmful, clinicians should parse out adaptive lactate signaling an intact stress response from lactate caused by hypoperfusion and cellular ischemia [15–17]. One clinical trial supports this hypothesis as administration of esmolol—pharmacologic blunting of the catecholamine response—reduced lactate concentrations in patients with septic shock [18]. Another study showed that increasing serum lactate in the earliest phases of sepsis, presumably due to sympathetic activation, was associated with reduced mortality [19].

Given these diametrically opposed positions regarding the deleterious effects of elevated serum lactate, we conducted this retrospective study to explore whether elevations of lactate in septic patients were driven primarily by abnormal blood pressure or by increased catecholamine activity. Because catecholamine levels are not measured directly during routine clinical care, we used patients’ presenting heart rates as a coarse proxy for their degree of inherent sympathetic activity.

## Materials and methods

### Patients

**Fig. 1:**
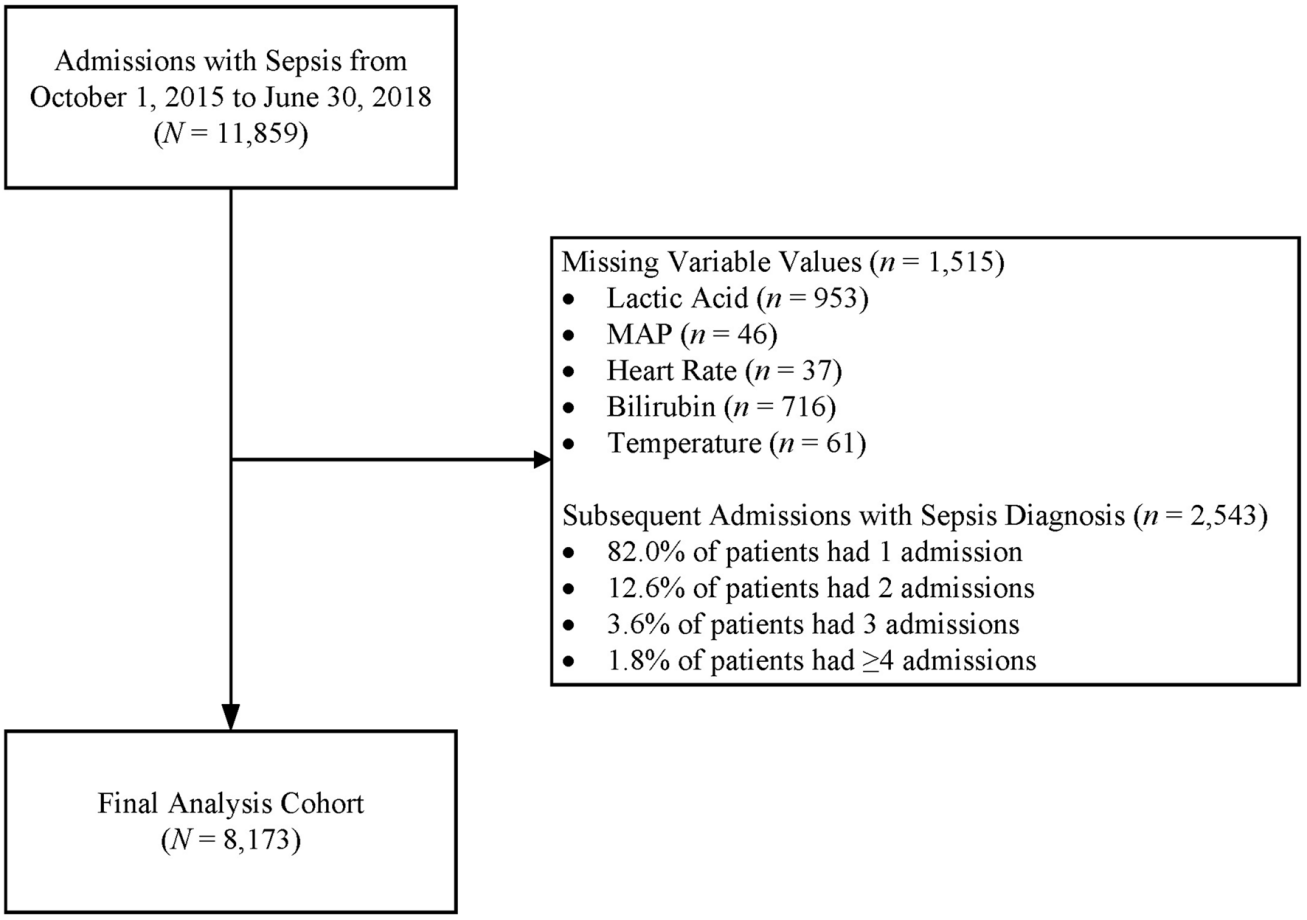
Schematic showing excluded admissions

After obtaining approval from Creighton University’s Institutional Review board, we retrospectively reviewed the charts of all hospital admissions between October 1, 2015, and June 30, 2018, that included an ICD-9/ICD-10 code for sepsis. A Structured Query Language (SQL) program was written to search for all patient visits in the clinical documentation system and that their final coding included a diagnosis code of sepsis, severe sepsis (with or without septic shock). Enrolled patients were admitted to any of the six Catholic Health Initiative hospitals in the Omaha, Nebraska, metropolitan area. We included only adult patients in this study: because the ages of majority are 19 years in Nebraska and 18 years in Iowa, this inclusion criterion varied geographically. Of the 11,859 identified sepsis admissions, 3686 were excluded for missing data or repeat sepsis admission, resulting in 8173 unique patients for analysis (see Fig. 1).

### Outcomes

The primary outcome was serum lactate level (in mmol/L) measured on admission. Secondary outcomes included in-hospital death and discharge from the intensive care unit (ICU).

### Covariates

All data abstracted/analyzed—including heart rate (HR), mean arterial blood pressure (MAP) and serum lactate—were collected at the time of presentation to the hospital, prior to the administration of fluids antibiotics, and/or vasopressors. When evaluating lactate level, MAP and HR were considered primary covariates. In separate analyses, we included MAP and HR as either continuous or dichotomous. When dichotomized, we used systemic inflammatory response syndrome (SIRS) criteria to stratify patients as hypotensive (MAP < 65 mm Hg) or normotensive (MAP ≥ 65 mm Hg) and as having low sympathetic drive (HR < 90 beats per minute [bpm]) or high sympathetic drive (HR ≥ 90 bpm). Secondary covariates included age, temperature, hepatic dysfunction on admission, history of heart failure, and diabetes. Age and temperature were mean centered prior to analysis, and hepatic dysfunction was defined as bilirubin concentration > 2 mg/dL.

## Statistical analysis

Depending on data distribution, continuous variables are presented as mean ± standard deviation or median and interquartile range; categorical variables are presented as frequency and percent alongside Agresti–Coull confidence intervals, when appropriate. Because serum lactate was severely positively skewed and heteroscedastic, it was modeled using an identity link and log-normal distribution of residuals. Two-way interaction effects were estimated to determine whether HR moderated the association between MAP and lactate. For categorical MAP and HR, all post hoc comparisons used the Tukey–Kramer adjustment to control Type I error rates. In-hospital death was modeled using logistic regression models; we evaluated both the moderation and mediation of HR on the effect of lactate on in-hospital death (see Additional file 1: Appendix for mediation analysis plan). The probability of discharge from the ICU was modeled using a Cox proportional-hazards model with ICU LOS censored upon patient death or at day 28. For all outcomes, the functional form of continuous predictors was assessed using Loess methods with nonlinearity modeled using piecewise linear effects as appropriate. The proportionality of hazards assumption was evaluated statistically via interactions with time. All regression-type models included fixed facility indicator variables to account for the clustering of patients within a facility. SAS v. 9.4 was used for all statistical analysis with *p* < 0.05 used to indicate statistical significance.

## Results

Table 1 summarizes the study participants’ demographic and clinical characteristics.

**Table 1:** Patient demographic and clinical characteristics (*N* = 8173)

Variable	Value
Male, *n* (%)	4062 (49.7)
Age, years, mdn [IQR]	67 [55–79]
BMI, kg/m^2^, mdn [IQR]	27.9 [23.5–33.9]
Lactate, mmol/L, mdn [IQR]	2.0 [1.3–2.9]
Heart rate, bpm, mean ± SD	100.4 ± 22.7
≤ 90, *n* (%)	2736 (33.5)
> 90, *n* (%)	5437 (66.5)
MAP, mm Hg, mean ± SD	93.2 ± 21.2
< 65, *n* (%)	709 (8.6)
≥ 65, *n* (%)	7470 (91.4)
Blood pressure, mmHg, mean ± SD	
Systolic	133.0 ± 30.5
Diastolic	73.3 ± 19.1
Bilirubin, mg/dL, mdn [IQR]	0.6 [0.4–1.0]
≤ 2, *n* (%)	7560 (92.5)
> 2, *n* (%)	613 (7.5)
Platelets, 10^9^/L, mdn [IQR]	219 [161–287]
Creatinine, mg/dL, mdn [IQR]	1.2 [0.9–1.8]
Temperature, °C, mdn [IQR]	36.9 [36.5–37.6]
History of heart failure, *n* (%)	1976 (24.2)
Diabetes, *n* (%)	2909 (35.6)
ICU LOS, days, mdn [IQR]	4 [2–7]
Hospital LOS, days, mdn [IQR]	5 [3–8]
Glasgow Coma Score, mdn [IQR]	15 [14,15]
Discharge disposition, *n* (%)	
Died	1041 (12.1)
Home	4368 (50.9)
Home healthcare	569 (6.6)
Long-term care	195 (2.3)
Skilled nursing	1736 (20.2)
Rehab facility	180 (2.1)
Other	496 (5.8)

### Serum lactate level

After adjusting for covariates, a statistically significant two-way interaction effect between categorical MAP and HR (interaction *p* = 0.004) was seen indicating that the lactate level for a given MAP category was different between the HR categories (Table 2). The highest lactate level was observed in patients with low MAP/high HR, whereas the lowest lactate level was observed in patients with high MAP/low HR. Lactate levels by group were statistically different from each other (each Tukey–Kramer adjusted *p* < 0.001) except for high HR/high MAP vs. low HR/low MAP (Tukey–Kramer adjusted *p* = 0.680).

**Table 2:** Adjusted model-estimated serum lactate levels by MAP and heart rate

Heart rate	< 65 mm Hg		≥ 65 mm Hg	
	*n*	mean [95% CI]	*n*	mean [95% CI]
Mean arterial pressure				
≤ 90 bpm	324	2.7 [2.5–2.9]	2412	2.2 [2.1–2.2]
> 90 bpm	379	3.6 [3.4–3.8]	5058	2.6 [2.5–2.6]

**Fig. 2:**
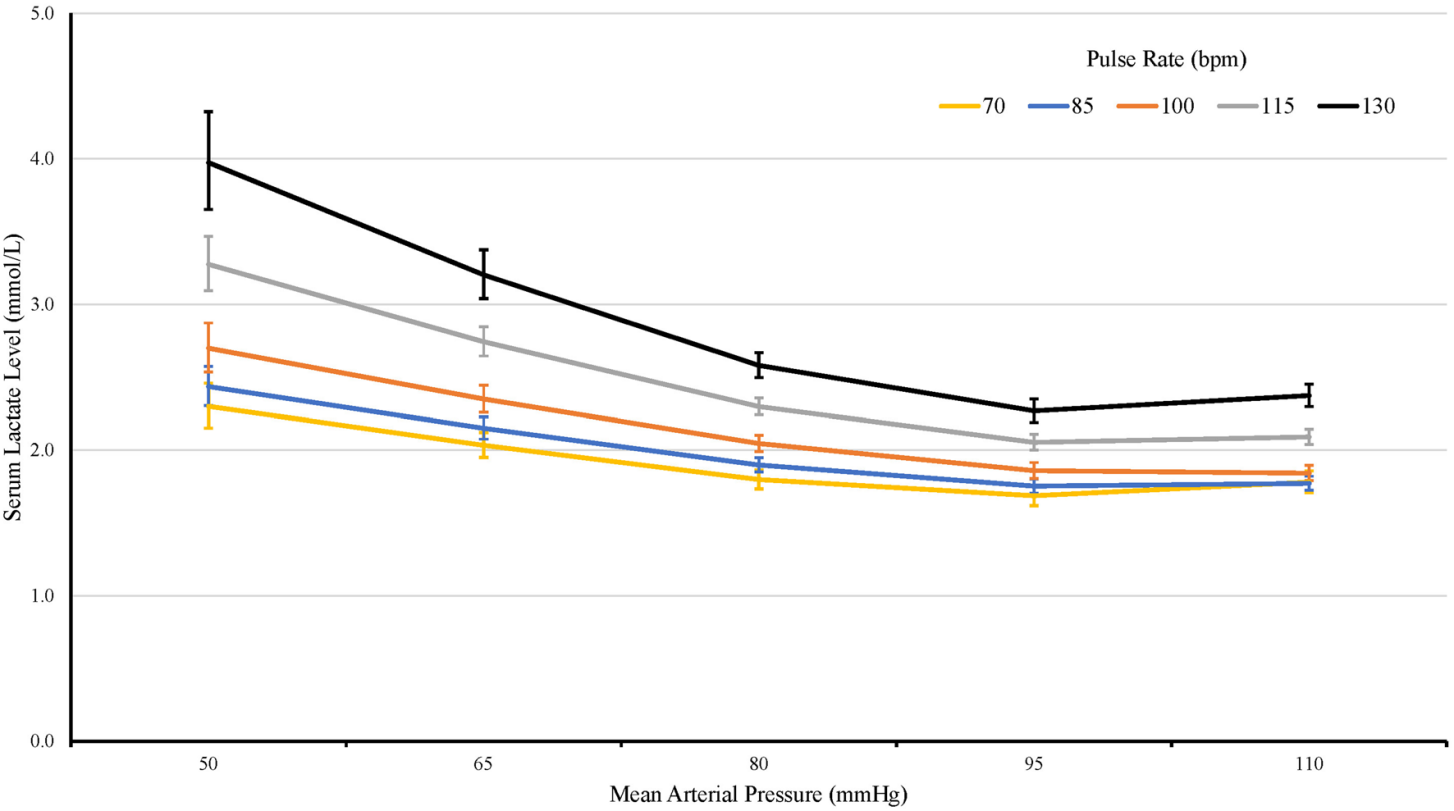
Model-estimated serum lactate level by mean arterial pressure and heart rate. Error bars represent 95% confidence intervals

In our cohort, the effect of continuous MAP and HR each had a piecewise linear association with lactate levels. Specifically, after adjusting for covariates, lactate level was 8.9% lower per 10 mmHg higher MAP until a MAP of 90 mmHg (95% CI 7.6% to 10.1%, *p* < 0.001), after which lactate was 1.9% higher per 10 mmHg higher MAP (95% CI 0.8% to 2.9%, *p* < 0.001). By contrast, after adjusting for covariates, lactate level was non-significantly 1.2% higher per 10-bpm higher HR until 95 bpm (95% CI 0.2% lower to 2.5% higher, *p* = 0.096), after which lactate increased 8.6% for every 10-bpm higher HR (95% CI 7.6% to 9.6%, *p* < 0.001). In the final model that combined the piecewise effects of MAP and HR, after adjusting for facility, statistically significant two-way interactions were indicated between MAP and HR (Fig. 2: see Additional file 2: Table S1 for full model results), such that the attenuating effect of higher MAP on lactate level was larger in patients with higher HR.

### In-hospital death

The overall in-hospital mortality rate for this cohort was 12.4% (95% CI 11.7% to 13.1%). As shown in Table 3, after adjusting for the covariates, every 1 mmol/L increase in lactate was associated with 20.8% higher odds of death (95% CI 17.4% to 24.2%, *p* < 0.001) and 10 bpm higher HR was associated with 6.0% higher odds of death (95% CI 2.6% to 9.4%, *p* < 0.001). By contrast, every 10 mmHg higher MAP was associated with 12.3% lower odds of death (95% CI 9.2% to 15.4%, *p* < 0.001). When evaluating whether HR moderated the odds of in-hospital death for higher lactate or MAP, the two-way interactions were not statistically significant (lactate-by-HR *p* = 0.082; MAP-by-HR *p* = 0.393) indicating that the odds of death reported above for lactate and MAP were constant irrespective of a patient’s HR. Further, HR did not mediate the association between lactate and death (see Additional file 1: Appendix for full results): although patients with higher HR had clinically higher lactate levels as reported above, patients with higher lactate levels did not have clinically higher HR (1 mmol/L higher lactate was associated with 1.8 bpm higher HR) which limited the ability of HR to mediate the association between lactate and death.

**Table 3:** Results for in-hospital death and time-to-ICU discharge

Variable	Unadjusted		Adjusted		
	OR [95% CI]	*p*	OR [95% CI]	Wald	*p*
In-hospital death					
Age (0 = 67; per 10 years)	1.46 [1.40–1.53]	< 0.001	1.55 [1.47–1.63]	271.41	< 0.001
Temperature (0 = 98.4)	0.86 [0.83–0.89]	< 0.001	0.90 [0.86–0.93]	32.35	< 0.001
Bilirubin > 2 mg/dL	2.70 [2.22–3.28]	< 0.001	2.33 [1.88–2.90]	58.21	< 0.001
History of heart failure	1.23 [1.06–1.43]	0.006	1.01 [0.86–1.19]	0.02	0.889
Diabetes	0.63 [0.55–0.73]	< 0.001	0.65 [0.56–0.76]	28.68	< 0.001
Lactate (per 1 mmol/L)	1.26 [1.23–1.29]	< 0.001	1.21 [1.17–1.24]	171.84	< 0.001
MAP (per 10 mmHg)	0.83 [0.80–0.85]	< 0.001	0.88 [0.85–0.91]	54.45	< 0.001
Heart rate (per 10 bpm)^a^					
≤ 95 bpm	0.87 [0.82–0.93]	< 0.001	–	–	–
> 95 bpm	1.07 [1.02–1.12]	0.005	–	–	–
Slope difference	–	< 0.001	–	–	0.058
Overall	–	–	1.06 [1.03–1.09]	12.31	< 0.001

Variable	Unadjusted		Adjusted		
	HR [95% CI]	p	HR [95% CI]	Wald	p
Discharge from the ICU					
Age (0 = 67; per 10 years)	0.95 [0.93–0.97]	< 0.001	0.95 [0.93–0.97]	27.70	< 0.001
Temperature (0 = 98.4)	1.07 [1.06–1.09]	< 0.001	1.06 [1.05–1.08]	58.95	< 0.001
Bilirubin > 2 mg/dL	0.65 [0.58–0.73]	< 0.001	0.70 [0.62–0.79]	35.30	< 0.001
History of heart failure	0.88 [0.82–0.94]	< 0.001	0.88 [0.82–0.95]	12.20	< 0.001
Diabetes	1.11 [1.04–1.18]	0.001	1.10 [1.03–1.17]	8.89	0.003
Lactate (per 1 mmol/L)	0.95 [0.93–0.96]	< 0.001	0.96 [0.95–0.98]	28.12	< 0.001
MAP (per 10 mmHg)	1.03 [1.01–1.04]	< 0.001	1.02 [1.01–1.03]	6.04	0.014
Heart rate (per 10 bpm)	1.01 [1.00–1.03]	0.024	0.99 [0.98–0.01]	1.62	0.203

Both the unadjusted and adjusted models included facility indicator variables as fixed effects to account for the clustering of patients within facilities.

^a^After adjusting for covariates, the slope difference in the log-odds of in-hospital death before vs. after HR of 95 was no longer statistically significant (*p* = 0.058); as such, the adjusted model includes a single overall effect of HR

*OR* odds ratio, *HR* hazard ratio, *MAP* mean arterial pressure

### Probability of ICU discharge

A total of 5145 (63.0%) patients spent time in the ICU during their hospital stay, of whom 4335 (84.3%) were discharged alive. After adjustment for covariates, 1 mmol/L higher lactate was associated with 4.0% lower likelihood of ICU discharge (95% CI 2.5% to 5.4%, *p* < 0.001), 10 mmHg higher MAP was associated with 1.7% higher likelihood of ICU discharge (95% CI 0.4% to 3.1%, *p* = 0.014), and 10 bpm higher HR was associated with a non-statistically significant 0.9% decreased likelihood of ICU discharge (95% CI 2.2% decrease to 0.5% increase, *p* = 0.203). We evaluated whether HR moderated the likelihood of ICU discharge for higher lactate or MAP. Although the lactate-by-HR interaction was not statistically significant (interaction *p* = 0.793; indicating that the effect of lactate was constant irrespective of HR), the association between MAP and ICU discharge was moderated by HR (interaction *p* = 0.023) such that higher MAP was associated with a higher likelihood of ICU discharge only in patients with HR at or above 97 bpm (Additional file 3: Figure S1).

### Sensitivity analyses: single admissions and the ICU cohort

Of the 8173 patients in our sample, 1506 (18.4%) had more than one hospital admission during the study period that included a sepsis diagnosis, for whom the median number of admissions was 2 [IQR: 2–3]. We expected that multiple sepsis admissions would be a proxy for severity; however, the mortality rate in patients with repeated admissions was 0.3% (vs. 15.2% in patients with only one admission; *p* < 0.001). We repeated all analyses excluding these patients and the results were nearly identical, albeit with slightly wider confidence intervals given the reduction in sample size. Finally, restricting the analysis to only the 5145 patients with an ICU admission resulted in replication of the pattern of results reported above for the overall cohort.

## Discussion

Lactate kinetics are multifaceted and often over-simplified in critical illness. An ongoing challenge to the primacy of lactate in sepsis has been the assertion that some degree of hyperlactatemia in septic patients results from catecholamine stimulation of ß2 receptors and that the resulting lactate (so-called Type B lactic acidosis) confounds the predictive utility of lactate in sepsis. Accordingly, our study attempted to explore this issue using data from a large cohort of sepsis patients and a relatively simplistic design. Our intent was to assess whether the effects of elevated lactate level in septic patients were affected by varying levels of systemic catecholamine activation (evidenced by higher presenting HR). Although our results support the hypothesis that patients with higher levels of sympathetic activity have higher levels of lactate, mortality was directly linked to elevations in lactate and was not affected by the degree of sympathetic activity.

Using accepted cut-points from the SIRS criteria for MAP and HR, we categorized septic patients as normotensive (MAP ≥ 65 mm Hg) or hypotensive (MAP < 65 mm Hg) and as having low sympathetic drive (HR < 90 bpm) or high sympathetic drive (HR ≥ 90 bpm). This allowed us to put septic patients into four groups: (1) hypotensive with low sympathetic drive; (2) hypotensive with high sympathetic drive; (3) normotensive with low sympathetic drive; and (4) normotensive with high sympathetic drive. Our hypothesis was that if both hypotension and sympathetic independently drive increased lactate levels, patients with both hypotension and high sympathetic drive would have the highest lactate levels, patients with hypotension or high sympathetic drive alone would have intermediate lactate levels, and patients with neither hypotension nor high sympathetic drive would have the lowest lactate levels. Our data precisely showed this relationship. Further, when holding MAP constant, we found that patients presenting with higher HR consistently had higher lactate levels compared to patients with lower HR (Fig. 2). These findings suggest that intrinsic sympathetic activity drive increases lactate levels. This relationship between HR and lactate was not affected by age, temperature, hepatic dysfunction, heart failure, and/or diabetes: when these factors were considered as covariates in statistical models, all inferences and the observed magnitude of the effects seen did not change.

Conventional wisdom regarding sepsis has focused on elevated lactate as a fundamental marker of tissue hypoxia due to distributive and/or hypovolemic shock. This has led to multiple disproven therapeutic recommendations including blood transfusions to maintain the hemoglobin > 10 mg/dL and using inotropes to drive the mixed venous oxygen saturation to > 70% among others. It is increasingly understood that in septic patients, excess lactate production through multiple avenues *and* reduced lactate clearance likely come into play—and that lactate production does not always signal physiologic aberrancy due to dysoxia [20–23].

However, any incremental lactate elevations due to increased catecholamines remained associated with an increased risk of death and were not less harmful as some have postulated. Consistent with numerous other studies, lactate was the strongest predictor of in-hospital death in this cohort of septic patients. Because we did not find a statistically significant interaction between HR and either lactate or MAP when looking at mortality or ICU discharge, our data do not suggest that increased HR (sympathetic drive) is protective. This is not entirely surprising and one could posit that our analysis is another example of the ‘squeezing the balloon’ analogy: any reduction in lactate production caused by an increase in MAP is replaced by a comparable amount of lactate due to the increased catecholamines needed to increase the HR to improve the MAP. Another alternate explanation suggests that lactate etiology (aerobic or anaerobic) is irrelevant and that lactate is a marker of a more severely dysregulated inflammatory response—a known maladaptive response [24]. Regardless, each of these hypotheses is consistent with the observation that elevated lactate levels are ominous even in the absence of shock [8]. Our study lends external validity to this association between lactate and mortality.

Our study supports the concept that hyperlactatemia in sepsis is more complex than simple anaerobic metabolism and that catecholamine stimulation can affect both MAP and lactate levels. One could propose that because lactate is not solely a marker of tissue hypoperfusion, intensivists should take pause in using lactate in isolation to guide resuscitation—an idea supported by a recent large, randomized, controlled trial which did not show benefit to using lactate kinetics to guide resuscitation. [25] As suggested by others, this subject deserves significant further study given the magnitude of the problem posed by septic shock coupled with the trend whereby lactate levels have evolved into a quality metric. [26] However, our finding that mortality was associated with increased lactate—and the lack of moderation/mediation of this relationship by HR—lactate appears to be an appropriate marker of severity of illness without consideration of the degree of sympathetic activity.

The biggest strengths of our study are the large sample size and the use of data from multiple hospitals, the majority of which were community-based. To maximize standardization despite using a retrospective dataset, all abstracted variables were objective measurements made at the time of admission. Because baseline clinical data and labs were used for analysis, we obviated the potential confounding effects of the most clinically important medications (i.e., beta-adrenergic agonists or blockers, sedatives) and other therapeutic interventions (i.e., mechanical ventilation) that could affect heart rate. We were also purposeful in including all patients with sepsis—not just those admitted to the ICU—as current sepsis alerts, treatment guidelines and/or quality metrics often include these individuals.

However, our study is not without significant limitations that must be considered when interpreting our results. The biggest barrier to drawing definitive conclusions from this study is our use of the HR as a surrogate for sympathetic activity as opposed to directly measuring catecholamine levels. Admittedly, HR is a very coarse measure of sympathetic activity and is potentially affected by multiple other factors including degree of fluid resuscitation, stroke volume, exogenous catecholamines, tachyarrhythmias, home medications (beta blockers, amiodarone, midodrine), fever, and comorbid illness (chronic hypertension). However, HR is uniformly available in real-time and is routinely used by clinicians at the bedside as a surrogate marker for sympathetic activation. Although we could effectively minimize the effects of some of these variables (fluid resuscitation, exogenous catecholamines) by collecting data at presentation prior to any therapy, other variables (tachyarrhythmia, comorbidity) could not be accounted for.

Another limitation was our reliance upon MAP as the sole marker of perfusion. We used MAP as our index of perfusion as it is a key component of the Surviving Sepsis Campaign and the ESICM guidelines. However, MAP does not consistently correlate with tissue perfusion at the microcirculatory level [27]. Other markers of tissue perfusion such as urine output, capillary refill, and central venous oxygen saturation were not reliably available across facilities. In addition to the biases inherent with a retrospective study design (selection bias, measurement error, confounding), we did not adjust for severity of illness via acuity scores (e.g., APACHE, SOFA) for two reasons: (1) APACHE score subsumes MAP and HR which precluded evaluation of the primary independent variables in this study and (2) acuity scores were not calculated for most patients cared for in the four community-based hospitals.

A definitive resolution of this issue would ultimately require further investigation—ideally prospective data with direct measurement of serum catecholamines, an assessment of presenting severity of illness, delineation of concurrent medications, and controlling for medical comorbidities. However, such an extensive endeavor should only be undertaken if we suspect that the effort might meaningfully alter clinical practice. Although imperfect, our data do not suggest any effect of sympathetic activity on the association between lactate and mortality. Accordingly, efforts to delineate this issue further do not appear justified.

## Conclusion

In this retrospective study, higher HR (used as a marker of sympathetic stimulation) was associated with higher lactate levels irrespective of MAP in septic patients. However, HR was not associated with either mortality or the likelihood of ICU discharge and lactate level was the most significant predictor of untoward outcomes. Because efforts to reverse hypotension in septic patients may simultaneously affect HR, the ultimate effects on lactate are complicated and potentially unpredictable. However, the strong association between lactate and mortality was not moderated or mediated by sympathetic activity. As such, lactate level is clearly a marker of severity of illness and mortality—whether it appears to be driven by hypotension or by sympathetic stimulation.

## Supplementary Information

**Additional file 1: Appendix S1.** Additional figures.

**Additional file 2:Table S1.** Serum lactate level: Final model results using continuous mean arterial pressure and heart rate using the entire cohort (used to create manuscript Fig. 2).

**Additional file 3: Figure S1.** Estimated hazard ratio for ICU discharge at specific HR for every 10 mm Hg higher MAP (shows MAP-by-HR interaction). Error bars represent 95% confidence intervals.

## Data Availability

The datasets used and/or analyzed during the current study are available from the corresponding author on reasonable request.

## Abbreviations

MAP: Mean arterial pressure
HR: Heart rate
SOFA: Sequential Organ Failure Assessment
SIRS: Systemic inflammatory response syndrome
ICU: Intensive care unit
