# Supplementary material for: Sympathetic stimulation increases serum lactate concentrations in patients admitted with sepsis: implications for resuscitation strategies
Source: Ann Intensive Care. 2021 Feb 5;11:24. doi: 10.1186/s13613-021-00805-9 (PMC7865043; doi:10.1186/s13613-021-00805-9)
Supplement: Supplementary file 1 — Additional file 1: Appendix S1. Additional figures. [file 13613_2021_805_MOESM1_ESM.docx]

**APPENDIX**

**Research Question**

Does heart rate (HR) mediate the relationship between serum lactate (in mmol/L) and in-hospital death?

**Brief Introduction to Mediation**

Mediation models generally consist of estimating three separate regression-type models, with the type of regression model dictated by the scale of measurement for the outcome. Several authors have described the four-step process for mediation models.^1-3^ For this study, to have mediation, we must 1) show that the serum lactate is associated with in-hospital death, 2) show that serum lactate is associated with HR, 3) show that HR is associated with in-hospital death, and 4) show that the association of serum lactate and in-hospital death is zero after controlling for HR. It is important to note that mediation is generally phrased in terms of clinical significance and relies less on statistical significance.^3^ Following this four-step process, the mediation models are shown below in equations 1, 2, and 3; they are also presented graphically in Figures A1 and A2, with arrows directed into outcomes. The effects of interest for our study are in red typeface.

|  | $\mathrm{logit}\left( \mathrm{Deat}h_{i}=1 \right)=c_{0}+c\left( \mathrm{Lactat}e_{i} \right)+c_{1}\left( {\mathrm{Facility}1}_{i} \right)+c_{2}\left( {\mathrm{Facility}2}_{i} \right)+$  $c_{3}\left( {\mathrm{Facility}3}_{i} \right)+c_{4}\left( {\mathrm{Facility}4}_{i} \right)+c_{5}\left( {\mathrm{Facility}5}_{i} \right)+$  $c_{6}\left( \mathrm{Age}_{i} \right)+c_{7}\left( \mathrm{Temp}_{i} \right)+c_{8}\left( {\mathrm{Bili}\mathrm{rubin}2}_{i} \right)+c_{9}\left( \mathrm{HxHF}_{i} \right)+$  $c_{10}\left( \mathrm{DM}_{i} \right)+c_{11}\left( \mathrm{MAP}_{i} \right)$ | (1) |
| --- | --- | --- |

|  | $\mathrm{HR}_{i}=a_{0}+a\left( \mathrm{Lactat}e_{i} \right)+a_{1}\left( {\mathrm{Facility}1}_{i} \right)+a_{2}\left( {\mathrm{Facility}2}_{i} \right)+a_{3}\left( {\mathrm{Facility}3}_{i} \right)+$  $a_{4}\left( {\mathrm{Facility}4}_{i} \right)+a_{5}\left( {\mathrm{Facility}5}_{i} \right)+a_{6}\left( \mathrm{Age}_{i} \right)+a_{7}\left( \mathrm{Temp}_{i} \right)+$  $a_{8}\left( {\mathrm{Bili}\mathrm{rubin}2}_{i} \right)+a_{9}\left( \mathrm{HxHF}_{i} \right)+a_{10}\left( \mathrm{DM}_{i} \right)+a_{11}\left( \mathrm{MAP}_{i} \right)+e_{i}$ | (2) |
| --- | --- | --- |

|  | $\mathrm{logit}\left( \mathrm{Deat}h_{i}=1 \right)=b_{0}+c^{'}\left( \mathrm{Lactat}e_{i} \right)+b\left( HR_{i} \right)+b_{1}\left( {\mathrm{Facility}1}_{i} \right)+b_{2}\left( {\mathrm{Facility}2}_{i} \right)+$  $b_{3}\left( {\mathrm{Facility}3}_{i} \right)+b_{4}\left( {\mathrm{Facility}4}_{i} \right)+b_{5}\left( {\mathrm{Facility}5}_{i} \right)+$  $b_{6}\left( \mathrm{Age}_{i} \right)+b_{7}\left( \mathrm{Temp}_{i} \right)+b_{8}\left( {\mathrm{Bili}\mathrm{rubin}2}_{i} \right)+b_{9}\left( \mathrm{HxHF}_{i} \right)+$  $b_{10}\left( \mathrm{DM}_{i} \right)+b_{11}\left( \mathrm{MAP}_{i} \right)$ | (3) |
| --- | --- | --- |

In the equations above, notice that HR is used both as a predictor in equation 1 and equation 3 as well as an outcome in equation 2. In Step 1, a logistic regression model, we estimate the unique effect of serum lactate on in-hospital death after adjusting for the covariates (path *c* in Figure A1 and equation 1; this effect is termed the *total effect*). Note that these initial results will be similar, but not identical to, those presented in the *In-Hospital Death* section of the manuscript’s Results section given that path *c* is not adjusted for HR. In Step 2, as shown in Figure A2 and equation 2, using a linear regression model, we estimate the unique effect of serum lactate on HR after adjusting for the covariates (path *a* in Figure A2). Finally, in Steps 3 and 4, we estimate a logistic regression model to obtain the unique effects of serum lactate and HR on in-hospital death after controlling for the covariates (paths *c’* and *b* in Figure A2; path *c’* is termed the *direct effect*).

By definition, HR would serve as a *complete mediator* of serum lactate if the direct effect of serum lactate on in-hospital death (path *c’* in Figure A2) is clinically nonsignificant after including HR in the model. Alternatively, HR would serve as a *partial mediator* if the direct effect of serum lactate on in-hospital death (path *c’* in Figure A2) showed a clinically significant, but not complete, reduction relative to the estimated total effect (path *c* in Figure A1).

Had all outcomes been measured on a continuous scale, the amount of mediation could be quantified directly by calculating the *indirect effect*, which is shown in equations 4 and 5 below. The indirect effect is the product of effects *a* and *b*.

|  | $total effect=direct effect+indirect effect$ | (4) |
| --- | --- | --- |

|  | $c=c^{'}+ab$ | (5) |
| --- | --- | --- |

However, in-hospital death is a binary outcome, which required a logistic regression model. In a logistic regression model, the residual variance is fixed at $\frac{\pi^{2}}{3}$ (assuming the scale parameter = 1). As such, the estimated coefficients in equations 1 and 3 (as well as equation 2) were on different scales. Therefore, to allow comparison between models and computation of the indirect effect, the coefficients of interest (i.e., paths *a*, *b*, *c*, and *c’*) needed to be rescaled. The procedure to create rescaled (aka, comparable) coefficients has been described previously, following which we can obtain an *approximate* indirect effect.^4^ The rescaling was completed within the software we used to estimate the mediation model.

**Statistical Analysis**

The mediation models were estimated by Model 4 within the PROCESS macro v. 3.3, which was called and run using SAS v. 9.4. The 95% bootstrapped confidence interval for the (approximate) indirect effect was based on 10,000 replications.^5^

**Results**

In Step 1 of the mediation model, 1 mmol/L higher serum lactate was associated with 22.0% higher odds of in-hospital death (*p* < .001; total effect path *c* in Figure A1 and equation 1). In Step 2 of the mediation model, 1 mmol/L higher serum lactate was associated with 1.9 bpm higher HR (*p* < .001; path *a* in Figure A2 and equation 2). In Step 3 of the mediation model, 1 bpm higher HR was associated with 0.1% higher odds of in-hospital death (*p* < .001; path *b* in Figure A2 and equation 3), whereas 1 mmol/L higher serum lactate was associated with 20.8% higher odds of in-hospital death (*p* < .001; path *c’* in Figure A2 and equation 3). After rescaling, the approximate indirect effect of HR was an estimated 0.01 (logit scale; bootstrap 95% CI: 0.01-0.02), with Sobel’s *p* < .001. Statistically, results indicated that HR partially mediated the association between lactate and in-hospital mortality; clinically, the amount of mediation was nonsignificant. Note that the ability of HR to mediate this association was severely limited given the lack of a clinically significant association between serum lactate and HR (i.e., very small path *a* effect). Full mediation results are presented in Tables A1 through A3.

**References**

1. Judd CM, Kenny DA. Process analysis: Estimating mediation in treatment evaluations. *Evaluation Review*. 1981;5:602-619.
2. James LR, Brett JM. Mediators, moderators and tests for mediation. *Journal of Applied Psychology*. 1984;69:307-321.
3. Baron RM, Kenny DA. The moderator-mediator variable distinction in social psychological research: Conceptual, strategic and statistical considerations. *Journal of Personality and Social Psychology*. 1986;51:1173-1182.
4. MacKinnon DP, Dwyer JH. Estimating mediated effects in prevention studies. *Evaluation Review.* 1993;17:144-158.
5. Hayes AF. *Introduction to mediation, moderation, and conditional process analysis: A regression-based approach*. 2^nd^ ed. New York: Guilford Press; 2017.

**Figure A1.** Step 1 of mediation testing to determine whether serum lactate has a unique association with in-hospital death after controlling for the covariates. Terms in parentheses map directly onto variables in Equations 1-3.

**Figure A2.** Steps 2-4 of mediation testing to determine whether serum lactate has a unique association with heart rate after controlling for the covariates and whether the effect of serum lactate on in-hospital death goes to zero after adding HR to the model. Terms in parentheses map directly onto variables in Equations 1-3.

**Table A1.** Unique effect of serum lactate on in-hospital death (Step 1)

|  |  |  |  |  | 95% CI for OR | |  |
| --- | --- | --- | --- | --- | --- | --- | --- |
| Variable | Path | Logit | SE | OR | Lower | Upper | *p* |
| Intercept | *c*_0_ | -1.67 | 0.24 | - | - | - | - |
| Facility |  |  |  |  |  |  |  |
| 1 | *c*_1_ | 0.33 | 0.17 | 1.40 | 0.99 | 1.97 | 0.057 |
| 2 | *c*_2_ | 0.24 | 0.20 | 1.28 | 0.86 | 1.90 | 0.230 |
| 3 | *c*_3_ | 0.34 | 0.18 | 1.40 | 0.98 | 2.00 | 0.067 |
| 4 | *c*_4_ | 0.00 | 0.18 | 1.00 | 0.70 | 1.43 | 0.985 |
| 5 | *c*_5_ | 0.10 | 0.19 | 1.10 | 0.75 | 1.61 | 0.623 |
| 6 | Reference | | | | | | |
| Age (0 = 67; per 1 year) | *c*_6_ | 0.04 | 0.00 | 1.04 | 1.04 | 1.05 | <.001 |
| Temperature (0 = 98.4) | *c*_7_ | -0.09 | 0.02 | 0.91 | 0.88 | 0.94 | <.001 |
| Bilirubin >2 mg/dL | *c*_8_ | 0.84 | 0.11 | 2.31 | 1.86 | 2.87 | <.001 |
| History of heart failure | *c*_9_ | -0.00 | 0.08 | 1.00 | 0.85 | 1.17 | 0.985 |
| Diabetes | *c*_10_ | -0.45 | 0.08 | 0.64 | 0.55 | 0.75 | <.001 |
| MAP (per 1 mmHg) | *c*_11_ | -0.01 | 0.00 | 0.99 | 0.98 | 0.99 | <.001 |
| Lactate (per 1 mmol/L) | *c* | 0.20 | 0.01 | 1.22 | 1.19 | 1.26 | <.001 |

*Note.* Values in the *Path* column map directly onto effects in Figures A1-A2 and Equations 1-3.

SE = standard error

OR = odds ratio

**Table A2.** Unique effects of serum lactate on heart rate (Step 2)

|  |  |  |  | 95% CI for Estimate | |  |
| --- | --- | --- | --- | --- | --- | --- |
| Variable | Path | Estimate | SE | Lower | Upper | *p* |
| Intercept | *a*_0_ | 94.19 | 1.07 | - | - | - |
| Facility |  |  |  |  |  |  |
| 1 | *a*_1_ | 1.01 | 1.10 | -1.14 | 3.17 | 0.357 |
| 2 | *a*_2_ | 2.81 | 1.27 | 0.32 | 5.31 | 0.027 |
| 3 | *a*_3_ | 2.48 | 1.15 | 0.22 | 4.75 | 0.032 |
| 4 | *a*_4_ | 0.46 | 1.12 | -1.73 | 2.64 | 0.683 |
| 5 | *a*_5_ | 4.88 | 1.20 | 2.52 | 7.23 | <.001 |
| 6 | Reference | | | | | |
| Age (0 = 67; per 1 year) | *a*_6_ | -0.29 | 0.01 | -0.32 | -0.27 | <.001 |
| Temperature (0 = 98.4) | *a*_7_ | 2.12 | 0.10 | 1.92 | 2.32 | <.001 |
| Bilirubin >2 mg/dL | *a*_8_ | -1.69 | 0.90 | -3.45 | 0.07 | 0.059 |
| History of heart failure | *a*_9_ | -2.40 | 0.56 | -3.51 | -1.30 | <.001 |
| Diabetes | *a*_10_ | -2.30 | 0.49 | -3.26 | -1.33 | <.001 |
| MAP (0 = 93; per 1 mmHg) | *a*_11_ | 0.14 | 0.01 | 0.11 | 0.16 | <.001 |
| Lactate (per 1 mmol/L) | *a* | 1.88 | 0.11 | 1.66 | 2.10 | <.001 |

*Note.* Values in the *Path* column map directly onto effects in Figures A1-A2 and Equations 1-3.

SE = standard error

**Table A3.** Unique effect of serum lactate and heart rate on in-hospital death (Steps 3 and 4)

|  |  |  |  |  | 95% CI for OR | |  |
| --- | --- | --- | --- | --- | --- | --- | --- |
|  | Path | Logit | SE | OR | Lower | Upper | *p* |
| Intercept | *b*_0_ | -2.16 | 0.28 | - | - | - | - |
| Facility |  |  |  |  |  |  |  |
| 1 | *b*_1_ | 0.34 | 0.18 | 1.41 | 1.00 | 1.99 | 0.051 |
| 2 | *b*_2_ | 0.24 | 0.20 | 1.27 | 0.85 | 1.90 | 0.236 |
| 3 | *b*_3_ | 0.33 | 0.18 | 1.40 | 0.97 | 2.00 | 0.070 |
| 4 | *b*_4_ | 0.01 | 0.18 | 1.01 | 0.71 | 1.44 | 0.947 |
| 5 | *b*_5_ | 0.08 | 0.20 | 1.08 | 0.74 | 1.58 | 0.697 |
| 6 | Reference | | | | | | |
| Age (0 = 67; per 1 year) | *b*_6_ | 0.04 | 0.00 | 1.05 | 1.04 | 1.05 | <.001 |
| Temperature (0 = 98.4) | *b*_7_ | -0.11 | 0.02 | 0.90 | 0.86 | 0.93 | <.001 |
| Bilirubin >2 mg/dL | *b*_8_ | 0.85 | 0.11 | 2.33 | 1.88 | 2.90 | <.001 |
| History of heart failure | *b*_9_ | 0.01 | 0.08 | 1.01 | 0.86 | 1.19 | 0.889 |
| Diabetes | *b*_10_ | -0.43 | 0.08 | 0.65 | 0.56 | 0.76 | <.001 |
| MAP (0 = 93; per 1 mmHg) | *b*_11_ | -0.01 | 0.00 | 0.99 | 0.98 | 0.99 | <.001 |
| Heart Rate (per 1 bpm) | *b* | 0.01 | 0.00 | 1.01 | 1.00 | 1.01 | 0.001 |
| Lactate (per 1 mmol/L) | *c’* | 0.19 | 0.01 | 1.21 | 1.17 | 1.24 | <.001 |

*Note.* Values in the *Path* column map directly onto effects in Figures A1-A2 and Equations 1-3.

SE = standard error

OR = odds ratio
