# Supplementary material for: Sympathetic stimulation increases serum lactate concentrations in patients admitted with sepsis: implications for resuscitation strategies
Source: Ann Intensive Care. 2021 Feb 5;11:24. doi: 10.1186/s13613-021-00805-9 (PMC7865043; doi:10.1186/s13613-021-00805-9)
Supplement: Supplementary file 2 — Additional file 2:Table S1. Serum lactate level: Final model results using continuous mean arterial pressure and heart rate using the entire cohort (used to create manuscript Fig. 2). [file 13613_2021_805_MOESM2_ESM.docx]

**Table S1.** Serum lactate level: Final model results using continuous mean arterial pressure and heart rate using the entire cohort (used to create manuscript Figure 2)

|  |  |  | 95% CI | |  |
| --- | --- | --- | --- | --- | --- |
| Variable | Estimate | SE | Lower | Upper | *p* |
| Intercept | 0.89 | 0.38 | 0.14 | 1.64 | - |
| Facility | 0.06 | 0.02 | 0.01 | 0.10 | 0.019 |
| 2 | 0.07 | 0.02 | 0.03 | 0.11 | <.001 |
| 3 | 0.00 | 0.02 | -0.03 | 0.04 | 0.846 |
| 4 | 0.08 | 0.02 | 0.04 | 0.13 | <.001 |
| 5 | 0.11 | 0.03 | 0.05 | 0.17 | <.001 |
| 6 | 0.89 | 0.38 | 0.14 | 1.64 | 0.020 |
| 1 | Reference | | | | |
| Age (0 = 67) | 0.00 | 0.00 | 0.00 | 0.01 | <.001 |
| Temperature (0 = 98.4) | -0.03 | 0.00 | -0.04 | -0.03 | <.001 |
| Bilirubin >2 mg/dL | 0.37 | 0.02 | 0.32 | 0.42 | <.001 |
| History of Heart Failure | -0.06 | 0.02 | -0.09 | -0.03 | <.001 |
| Diabetes | 0.08 | 0.01 | 0.05 | 0.11 | <.001 |
| MAP ≤90 mm Hg | -0.01 | 0.00 | -0.02 | 0.00 | 0.120 |
| MAP >90 mm Hg | 0.02 | 0.00 | 0.01 | 0.03 | 0.001 |
| HR ≤95 bpm | 0.00 | 0.00 | -0.00 | 0.01 | 0.365 |
| HR >95 bpm | 0.02 | 0.00 | 0.01 | 0.03 | <.001 |
| MAP ≤90 by HR ≤95 | 0.00 | 0.00 | -0.00 | 0.00 | 0.906 |
| MAP ≤90 by HR >95 | -0.00 | 0.00 | -0.00 | 0.00 | 0.001 |
| MAP >90 by HR ≤95 | -0.00 | 0.00 | -0.00 | 0.00 | 0.001 |
| MAP >90 by HR >95 | 0.00 | 0.00 | 0.00 | 0.00 | <.001 |

*Note*. All estimates are on the natural log scale given the use of lognormal distribution of residuals (scale = 0.3371, SE = 0.005279). Standard errors (SE) reported as 0.00 were all estimated and >0.0049; they appear as 0.00 due to rounding.

MAP = mean arterial pressure, HR = heart rate, SE = standard error.
