# Supplementary figures and images for: Sympathetic stimulation increases serum lactate concentrations in patients admitted with sepsis: implications for resuscitation strategies
Source: Ann Intensive Care. 2021 Feb 5;11:24. doi: 10.1186/s13613-021-00805-9 (PMC7865043; doi:10.1186/s13613-021-00805-9)

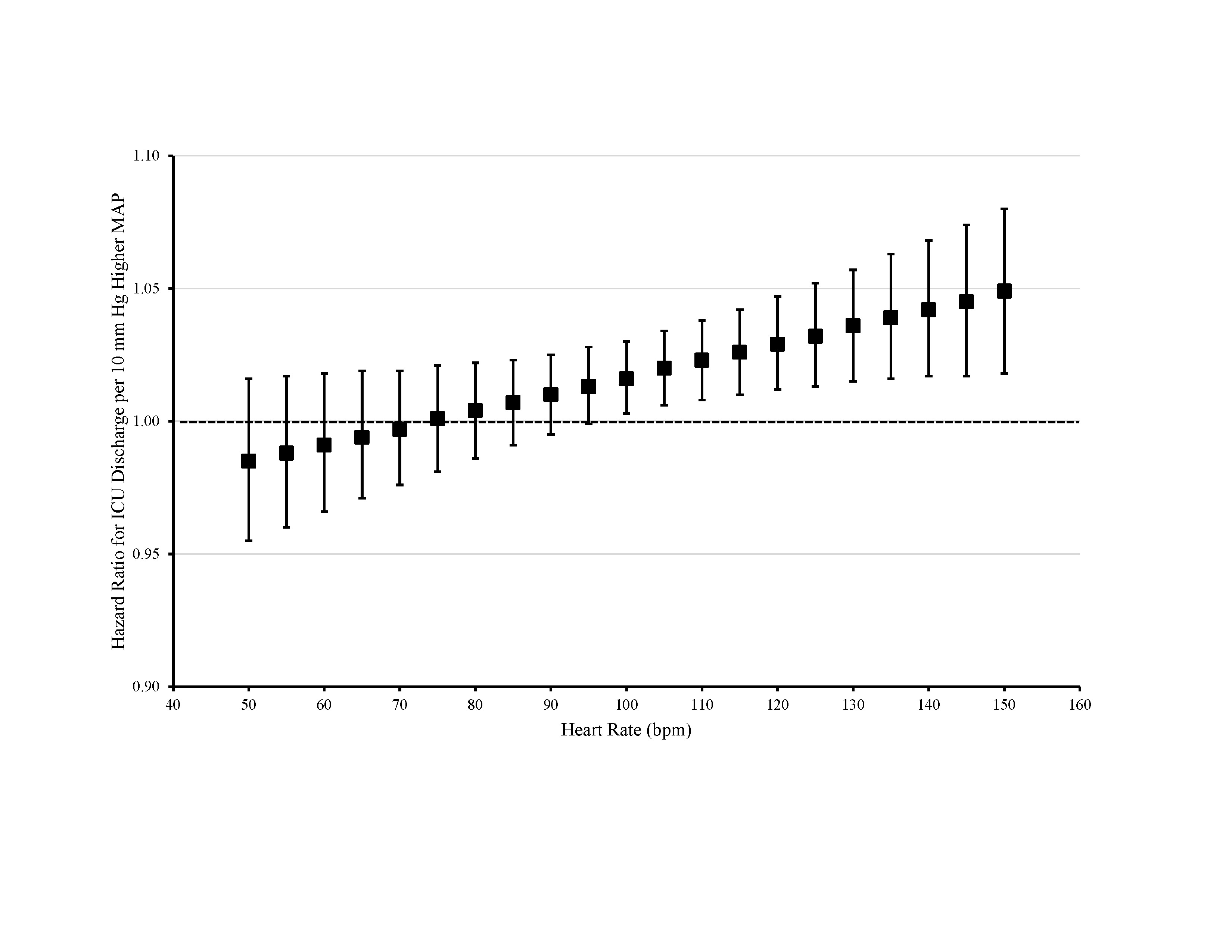

Supplement: Supplementary file 3 — Additional file 3: Figure S1. Estimated hazard ratio for ICU discharge at specific HR for every 10 mm Hg higher MAP (shows MAP-by-HR interaction). Error bars represent 95% confidence intervals. [file 13613_2021_805_MOESM3_ESM.jpg]
